# Mambalgin-2 Inhibits Growth, Migration, and Invasion of Metastatic Melanoma Cells by Targeting the Channels Containing an ASIC1a Subunit Whose Up-Regulation Correlates with Poor Survival Prognosis

**DOI:** 10.3390/biomedicines9101324

**Published:** 2021-09-26

**Authors:** Maxim L. Bychkov, Artem V. Kirichenko, Mikhail A. Shulepko, Irina N. Mikhaylova, Mikhail P. Kirpichnikov, Ekaterina N. Lyukmanova

**Affiliations:** 1 Shemyakin-Ovchinnikov Institute of Bioorganic Chemistry, Russian Academy of Sciences, 119997 Moscow, Russia; maksim.bychkov@gmail.com (M.L.B.); bittert@mail.ru (A.V.K.); mikhailshulepko@gmail.com (M.A.S.); kirpichnikov@inbox.ru (M.P.K.); 2 Moscow Institute of Physics and Technology (State University), 141701 Dolgoprudny, Russia; 3 Federal State Budgetary Institution “N.N. Blokhin National Medical Research Center of Oncology”, Ministry of Health of Russia, 115548 Moscow, Russia; irmikhaylova@gmail.com; 4 Faculty of Biology, Lomonosov Moscow State University, 119234 Moscow, Russia

**Keywords:** melanoma, acid-sensing ion channels, mambalgin-2, cancer, media acidification

## Abstract

Melanoma is an aggressive cancer characterized by the acidification of the extracellular environment. Here, we showed for the first time that extracellular media acidification increases proliferation, migration, and invasion of patient-derived metastatic melanoma cells and up-regulates cell-surface expression of acid-sensitive channels containing the ASIC1a, α-ENaC, and γ-ENaC subunits. No influence of media acidification on these processes was found in normal keratinocytes. To control metastatic melanoma progression associated with the ASIC1a up-regulation, we proposed the ASIC1a inhibitor, -mambalgin-2 from *Dendpoaspis polylepis* venom. Recombinant analog of mambalgin-2 cancelled acidification-induced proliferation, migration, and invasion of metastatic melanoma cells, promoted apoptosis, and down-regulated cell-surface expression of prooncogenic factors CD44 and Frizzled 4 and phosphorylation of transcription factor SNAI. Confocal microscopy and affinity purification revealed that mambalgin-2 interacts with heterotrimeric ASIC1a/α-ENaC/γ-ENaC channels on the surface of metastatic melanoma cells. Using the mutant variant of mambalgin-2 with reduced activity toward ASIC1a, we confirmed that the principal molecular target of mambalgin-2 in melanoma cells is the ASIC1a subunit. Bioinformatic analysis confirmed up-regulation of the ASIC1 expression as a marker of poor survival prognosis for patients with metastatic melanoma. Thus, targeting ASIC1a by drugs such as mambalgin-2 could be a promising strategy for metastatic melanoma treatment.

## 1. Introduction

Melanoma is a very heterogeneous form of cancer, characterized by severe proliferation, invasion, and drug resistance [1]. All these processes are accompanied by induction of hypoxia and elevated extracellular acidification [2,3]. Aerobic glycolysis, hyperactivation of the pentose phosphate pathway, hypoxia, and glutamine metabolism in dividing cancer cells lead to acidosis of the tumor microenvironment to pH ~6.5 instead of ~7.4 in normal conditions [4], while the intracellular pH remains neutral. Melanoma cells adapt to the acidic extracellular environment by the increase of glycolytic activity and hyperexpression of proton exporters (such as Na^+^/H^+^ exchanger or monocarboxylate transporter) to stabilize intracellular pH [4]. In general, acidification of the extracellular microenvironment is harmful to cells as it can drive apoptosis, however in tumor cells it can promote proliferation and migration [4,5]. After adaptation to the acidic conditions, melanoma cells exhibit the upregulation of signaling pathways important for tissue remodeling, cell cycle progression, proliferation, and motility, even if the extracellular pH returns to physiological values [6,7]. Another facet of the melanoma extracellular media acidification is induction of the epithelial-mesenchymal transition (EMT). For example, low pH leads to up-regulation of mesenchymal markers (N-cadherin, Vimentin) and transcription factors (Twist, NF-κB), as well as enhanced MMP-9 activity associated with invasiveness increase [7]. Up-regulation of growth and migration factors of acid-adapted melanoma cells leads to enhanced metastasis formation even at non-acidic physiological environment, and the cells cultivated in the acidic environment promote invasiveness of non-acidic cells in vitro [4,7]. In addition, long-term cultivation of melanoma cells in the acidic media causes up-regulation of CD133, SOX2, and other molecules, important for self-renewal of melanoma cells [8]. Thus, acidification of the extracellular media may drive growth, migration, and invasion of melanoma cells; and targeting of the molecules, responsible for melanoma cell adaptation to the acidic conditions can be a perspective strategy for melanoma therapy.

One of the most important pH sensors are acid-sensitive ion channels (ASICs)—cation channels from the degenerin/epithelial Na^+^ channel (DEG/ENaC) superfamily—which are expressed on the cell-surface membrane as homo- or heterotrimers and are activated by the extracellular acidification. ASICs have different functions in the nervous system in physiological and in pathological processes. In the central nervous system, ASIC1 channels participate in neuroplasticity, regulation of fear behaviors, learning, memory functions, and pain sensation [9], however, there are also the data about the ASIC1 participation in leukemia [10]**,** glioma [11], breast [12], hepatocellular [13]**,** and lung cancer [5] progression and induction of EMT [14,15]. Recently, the ASIC1 expression was found in melanoma and non-melanocytic skin cancers [16]**,** but the ASIC1 role in melanoma progression, as well as the possibility of ASIC1 targeting by selective inhibitors as a new therapeutic strategy for melanoma therapy were not investigated yet.

Inhibitors of ASIC1a such as amiloride and psalmotoxin down-regulate growth of GBM [17,18] and carcinoma [5,12] cells, but these drugs demonstrate irreversibility of binding and low selectivity [17,18,19,20]. Mambalgin-2 from *Dendroaspis polylepis* is known as a selective and reversible inhibitor of the ASIC1a channels [21,22]. Recently, we reported that recombinant mambalgin-2 effectively inhibits the acidification-induced growth of leukemia [10] and GBM cells [23], including primary cells derived from a patient with GBM by interaction with ASIC1a and induces cell cycle arrest and apoptosis. Here, we investigated the influence of the extracellular acidification on patient-derived metastatic melanoma cell growth, migration, and invasion and revealed for the first time the up-regulation of the channels containing the ASIC1a, α-ENaC, and γ-ENaC subunits in metastatic melanoma cells under acidic conditions. Treatment by mambalgin-2 led to down-regulation of growth, migration, and invasion of metastatic melanoma cells, induction of apoptosis, and inactivation of a number of factors related with migration, invasion and stemness. Finally, we studied the targets of mambalgin-2 in metastatic melanoma cells and performed bioinformatic analysis of expression of different members of the DEG/ENaC family in patients with various types of melanomas. Altogether, our data show that ASIC1a can be a perspective molecular target for metastatic melanoma treatment and mambalgin-2 may be considered as a promising prototype for the design of new anti-melanoma drugs.

## 2. Materials and Methods

### 2.1. Materials

Recombinant mambalgin-2 and its mutant variant with the Leu32Ala substitution were produced in *E. coli*, as described previously [24]. The purity and homogeneity of the recombinant proteins (>95%) were confirmed by HPLC, MALDI-MS, and SDS-PAGE. Disulfide bond formation was confirmed in the reaction with Ellman’s reagent (Sigma-Aldrich, Saint-Louis, MO, USA). The correct spatial structure of the recombinant proteins was confirmed by 1D ^1^H-NMR-spectroscopy. Fluorescent labeling of mambalgin-2 with CF647 dye was done using the CF-647 Protein Labeling Kit (Sigma-Aldrich).

### 2.2. Real-Time PCR

Total RNA was isolated with the Bio-Rad Aurum RNA isolation kit (Bio-Rad, Hercules, CA, USA) according to manufacturer instructions. cDNA was synthesized by the Mint reverse transcriptase kit (Evrogen, Moscow, Russia). After that, qPCR was performed with ready to use SYBR Green HS mix (Evrogen) and primers specific to the *ACCN2, ACCN1, ACCN3, ACCN4, SCNN1A,* and *SCNN1G* genes (Table 1) using the Roche LightCycler 96 amplifier (Roche, Basel, Switzerland).

Data were analyzed by the ∆∆Ct method and LightCycler SW software (Roche), and the gene expression was normalized to the expression of *β-ACTIN*, *GPDH,* and *RPL13a* housekeeping genes.

### 2.3. Cell Cultivation and Proliferation Assay

Metastatic skin melanoma mel P cells were obtained from a patient of Federal State Budgetary Institution “N.N.Blokhin National Medical Research Center of Oncology” of the Ministry of Health of Russia (Moscow, Russia) after informed consent and characterized previously [25,26]. The cells were deposited in the Russian Vertebrate cell culture collection (#688D, St-Petersburg, Russia), where were taken from. Mel P cells were grown in RPMI-1640 media (PanEco, Moscow, Russia) supplemented with 10% fetal calf serum and 1% penicillin/streptomycin. Human immortalized oral Het-1A keratinocytes (ATCC, Manassas, VA, USA) were cultivated in BEB medium (Lonza, Bazel, Switzerland). Before cell subculturing and performing experiments, the culture flasks and plates were pre-coated with a mixture of 0.01 g/L fibronectin (Sigma-Aldrich), 0.03 g/L bovine collagen type I (Sigma-Aldrich), and 0.01 g/L bovine serum albumin (Sigma-Aldrich) dissolved in the corresponded culture medium. Cells were maintained at 37 °C in a humidified atmosphere with 8% CO_2_. All types of cells were subcultured twice per week. The cells were passaged for no more than 40 times and regularly tested for absence of mycoplasma contamination by the PCR kit (Mycoreport, Evrogen).

For investigation of the media acidification influence on the mel P cells morphology, the cell medium was supplemented with 25 mM HEPES, pH 6.5 (acidic medium), while the cell medium with pH 7.4 is called throughout the manuscript as a normal medium. The chosen pH value for the acidic medium corresponds to the pH value of melanoma lesions [4] and was not toxic to mel P or Het-1A cells. To assay the media acidification influence on the cell growth, cells were seeded in 6-well culture plates (2 × 10^5^ cells per well) in the normal or acidic medium and were incubated for 96 h with the media change by the normal or acidic medium, respectively, every 48 h. After that, the photo of the cells were taken using CloneSelect Imager (80× magnification, Molecular Devices, San Jose, CA, USA), and the cell viability and cell cycle were analyzed (see below). Then, cells were kept in the acidic or normal media and cultivated as usual. For analysis of the acidification influence on the cell duplication time, the cells were seeded in 25 cm^2^ cell culture flasks (5 × 10^5^ cells/flask), detached every 24 h by 0.5% trypsin-EDTA solution (PanEco), and counted using the Cell Drop cell counter (DeNovix, Wilmington, DE, USA). After 96 h of cultivation, the growth curve was obtained by linear regression option of GraphPad Prism 8.0 (GraphPad Software, San Diego, CA, USA).

To study the mambalgin-2 influence on the cell proliferation, the cells were seeded in 96-well cell culture plates (5 × 10^3^ cells/well) in the acidic media and grown for 24 h. Thereafter, mambalgin-2 (from the 10 mM stock solution in 100% DMSO) was dissolved in the acidic medium and added to the cells at concentrations from 10**^−^**^10^ to 10**^−^**^5^ M for further incubation during 72 h without the media change. The maximal DMSO concentration did not exceed 0.1%. The added DMSO did not influence the cell growth as was established in additional experiments.

To analyze cell viability, we used the WST-1 colorimetric test as described earlier [27]. Briefly, WST-1 (water-soluble tetrazolium salt 1; Santa Cruz, Dallas, TX, USA) and 1-m-PMS (1-methoxy-5-methylphenazinium methyl sulfate, Santa Cruz) were added to the cells in concentrations of 0.25 mM and 5 μM, respectively, for 1 h, and formation of the colored product was measured at 450 nm with background subtraction at 655 nm on microplate reader Bio-Rad 680 (Bio-Rad, Hercules, CA, USA). The data were normalized to averaged read-out from the control wells, containing cells without added compounds. The concentration-effect curves were fitted in GraphPad Prism 8.0 (GraphPad Software). To visualize the mambalgin-2 influence on a colony formation the crystal violet assay was performed. Briefly, cells were seeded in 24-well plates (15 × 10^4^ cells/well), grown for 24 h, treated with mambalgin-2 for 72 h, washed in phosphate-buffered saline (PBS), fixed in 70% ethanol solution, stained with 1% crystal violet solution, and washed in PBS 3 times. Thereafter, the pictures of the wells were taken for the analysis.

### 2.4. Cell Cycle Analysis

For analysis of the acidification influence on the cell cycle, the mel P cells were seeded in 6-well culture plates (2 × 10^5^ cells per well) in the normal or acidic medium and grown for 96 h with the media change every 48 h. For analysis of the mambalgin-2 influence on the cell cycle, mel P cells were seeded in 6-well culture plates (2 × 10^5^ cells per well) in the acidic medium, grown for 24 h, and incubated with 1 μM mambalgin-2 for 72 h without the media change. Then the cells were detached from the wells by 0.5% trypsin-EDTA, washed with Earl balanced salt solution (EBSS), and fixed in ice-cold 70% ethanol for 24 h at −20 °C. After fixation, the cells were washed twice by EBSS, and DNA was extracted by 5 min incubation with the DNA extraction buffer (200 mM Na_2_HPO_4_ with 0.004% Triton X-100, pH 7.8). Then the cells were washed with EBSS, resuspended in the DNA staining solution (EBSS, 50 mg/mL propidium iodide, 0.2 mg/mL DNAse free RNAse A), and analyzed by the Attune NxT flow cytometer (Life Technologies, Carlsbad, CA, USA). The data were analyzed using the Attune NxT flow cytometer Software (Life Technologies).

### 2.5. Flow Cytometry and Protein Expression/Phosphorylation

For investigation of the media acidification influence on the ASIC1a, α-ENaC, and γ-ENaC subunits expression, cells were seeded in 6-well plates (2 × 10^5^ cells per well) in the normal or acidic media and grown for 96 h with the media change every 48 h. After that, cells were fixed in 4% PFA for 15 min and sequentially incubated with antibodies for ASIC1a (sheep, 1:500, ABIN350049, Antibodies-Online, Aachen, Germany), α-ENaC (rabbit, 1:500, ABIN1841945, Antibodies-Online) or γ-ENaC (mouse, 1:500, ABIN1865926, Antibodies-Online). After that, cells were washed in PBS and incubated with TRITC-conjugated anti-sheep (1:500, 713-025-003, Jackson Immunoresearch, West Grove, PA, USA), AlexaFluor488-conjugated anti-rabbit (1:500, 611-545-215, Jackson Immunoresearch) or AlexaFluor488-conjugated anti-mouse (1:500, 715-545-150, Jackson Immunoresearch) antibodies, respectively, for 1 h, washed in PBS, and the receptors expression was analyzed using the Beckman Coulter Cytoflex flow cytometer (Beckman Coulter, Brea, CA, USA). The data was analyzed by the Cytoflex 2.4. software (Beckman Coulter).

For analysis of the media acidification influence on the expression and phosphorylation of other cellular receptors or intracellular messengers, cells were seeded at 24-well plates (15 × 10^3^ cells per well) in the normal or acidic medium and were cultivated for 96 h with the media change every 48 h. For analysis of the mambalgin-2 influence on the protein expression and phosphorylation, cells were seeded at 24-well plates (15 × 10^3^ cells per well) in the acidic medium, cultivated for 24 h, and then incubated with 1 μM mambalgin-2 for 72 h without the media change. After incubations, cells were detached by Versene solution, fixed in 4% PFA for 15 min, and incubated with the primary mouse antibodies for CD44 (1:500, ABIN969026, Antibodies-Online) and CD133 (1:500, ABIN6559815, Antibodies-Online), primary rabbit antibodies for Frizzled 4 (1:250, ABIN5693200, Antibodies-Online) and SNAI1 (pSer246) (1:250, ABIN6256209, Antibodies-Online), or with in-house biotinylated antibodies for NFκB p65 (pSer536) (1:100, AHP1342, Bio-Rad) and STAT3 (pTyr705) (1:100, VMA00899, Bio-Rad). Before incubation with the primary antibodies for SNAI (pSer246), NFκB p65 (pSer536), and STAT3 (pTyr705), mel P cells were permeabilized by 0.1% Triton X100 in PBS for 15 min at room temperature. After incubation with the primary antibodies, cells were washed and incubated with the secondary TRITC-conjugated anti-mouse antibodies (115-025-062, Jackson Immunoresearch), TRITC-conjugated anti-rabbit antibodies (611-025-215, Jackson Immunoresearch), or with streptavidin-phycoerythrin (STAR4A, Bio-Rad) for 1 h. After that, cells were washed by PBS and analyzed using the Attune NxT flow cytometer (Life Technologies). At least 5 × 10^3^ of cells in the gate were analyzed. The data were normalized to read-out from cells stained only by the secondary antibodies or streptavidin-PE. The data were analyzed by the Attune NxT Software (Life Technologies). For all flow cytometry assays, the Median Fluorescence intensity (MFI) of cells stained with the primary and secondary antibodies was normalized to MFI of cells, stained with only by the secondary antibodies.

### 2.6. Wound Healing (Scratch) and Invasion Assays

The in vitro wound healing (scratch) assay was performed as described earlier [28] with some changes. In brief, mel P or Het-1A cells were seeded in 96-well cell culture plates in the acidic medium (5 × 10^4^ cells/well) and grown for 24 h. Then the media from the wells was changed to serum-free media to minimize cell proliferation. After 8 h the wells were scratched with a sterile 10 μL pipette tip. Then, the cells were washed with PBS, treated with 10 µM mambalgin-2, and pictures were taken after 0 and 24 h at 20× magnification using the CloneSelect Imager (Molecular Devices). The center of the plate was marked as a central reference point to ensure recording of the same area during the assay. Digital images were taken, and the scratch area was quantified using the ImageJ (NIH, Bethesda, MD, USA) and MS Excel software (Microsoft, Seattle, WA, USA) by measurement% of the scratch surface, occupied by migrating cells. In each experiment, the duplicate measurements have been averaged.

For investigation of cell invasion, mel P cells were cultivated in the normal or acidic media for 96 h with the media change every 48 h and then the Abcam migration/chemotaxis assay kit (ab235694, Abcam, Cambridge, UK) based on cell migration through the membrane with 8 µm pores was used for invasion measurement. For investigation of the mambalgin-2 influence on invasion, mel P cells cultivated in the acidic media for 96 h were seeded in migration chambers in 24-well plates (2 × 10^5^ cells per well) in the acidic media and incubated with 10 μM mambalgin-2 for 72 h without the media change. The cells migrated through the 8-µm pores were photographed (100 × magnification, Micro-Med I-LUM microscope, St-Petersburg, Russia), and the cell number was analyzed by the WST-1 test.

### 2.7. Confocal Microscopy

To study the mambalgin-2 molecular targets in mel P cells, the cells were seeded on round glasses in 24-well plates (15 × 10^4^ cells/well) in the acidic medium and grown for 24 h. Thereafter, cells were fixed with 4% PFA for 15 min, blocked by 2% bovine serum albumin solution in PBS for 1 h and incubated with the primary antibodies for ASIC1a (sheep, 1:500, ABIN350049, Antibodies-Online), α-ENaC (rabbit, 1:500, ABIN1841945, Antibodies-Online) or γ-ENaC (mouse, 1:500, ABIN1865926, Antibodies-Online) subunits. After that, cells were incubated with the TRITC-conjugated anti-sheep (1:500, 713-025-003, Jackson Immunoresearch), AlexaFluor488-conjugated anti-rabbit (1:500, 611-545-215, Jackson Immunoresearch) or AlexaFluor488-conjugated anti-mouse (1:500, 715-545-150, Jackson Immunoresearch) antibodies and with mambalgin-2 labeled by CF647 dye. Cell nuclei were stained by Hoechst 33342, cells were mounted in the ProLong Gold antifade mounting medium (Life Technologies) and observed using the Carl Zeiss LSM710 inverted confocal microscope (Carl Zeiss, Jena, Germany) using × 60 (1.4) oil-immersion objective.

### 2.8. Affinity Purification and Western Blotting

Mambalgin-2 (1 mg/mL) was coupled to NHS-activated Sepharose 4 Fast Flow (Cat #17-0906-01, GE Healthcare, Chicago, IL, USA) according to the manufacturer’s manual. The resin blocked by 500 mM ethanolamine without any protein coupled was used as a negative control. The membrane fraction of mel P cells (5 × 10^7^ cells per sample) was solubilized in 2% Triton X-100 (Cat# A4975, Panreac, Barcelona, Spain), diluted 10 times with TBS buffer (100 mM TRIS, 150 mM NaCl, pH 8.0) and incubated with the resin for 16 h at 4 °C in TBS. After that, non-specifically bound proteins were sequentially washed out from the resin with 5 volumes of TBS, 5 volumes of TBS + 1 M NaCl + 0.5% Triton X-100, and 5 volumes of TBS + 0.5% Triton X-100. The specifically bound proteins were eluted by 5 volumes of 200 mM Glycine (pH 2.6), diluted in the loading buffer (120 mM Tris-HCl, 20% [*v/v*] glycerol, 10% [*v/v*] mercaptoethanol, 4% [*w/v*] sodium dodecyl sulfate, and 0.05% [*w/v*] bromophenol blue, pH 6.8), submitted to gel electrophoresis, blotted onto nitrocellulose membranes (GE Healthcare) and blocked for 2 h in 5% skim milk (Sigma-Aldrich) in TBS (50 mM Tris, 150 mM NaCl, pH 7.4) buffer + 0.1% Tween-20 (Applichem, Darmstadt, Germany). The membranes were incubated overnight at 4 °C with primary antibodies for ASIC1a (sheep, 1:1000, ABIN350049, Antibodies-Online), ASIC3 (rabbit, 1:1000, ABIN3187736, Antibodies-Online), α-ENaC (rabbit, 1:1000, ABIN1841945, Antibodies-Online) or γ-ENaC (mouse, 1:1000, ABIN1865926, Antibodies-Online); washed 3 times with TBS + 0.1% Tween-20 and incubated with HRP-conjugated secondary anti-sheep antibody (1:5000, 713-035-003, Jackson Immunoresearch) in the case of ASIC1a, anti-rabbit antibody (1:5000, 111-035-003, Jackson Immunoresearch) in the cases of ASIC3 and α-ENaC or anti-mouse antibody (1:5000, 715-035-150, Jackson Immunoresearch) in the case of γ-ENaC for 1 h (20 °C). After that, membranes were washed 4 times with TBS + 0.1% Tween-20, and an HRP signal was detected by ECL substrate (Bio-Rad) with the ImageQuant LAS 500 chemidocumenter (GE Healthcare).

### 2.9. Analysis of Phosphatidylserine Externalization

To investigate apoptosis in mel P cells, we used Annexin V for detection of the phosphatidylserine externalization—one of the early apoptosis markers. Briefly, cells were seeded on 6-well plates (2 × 10^5^ cells/well) and incubated with 10 μM of mambalgin-2 for 72 h. After incubation, the cells were detached by the Versene solution and washed in the annexin-binding buffer (V13246, Thermo Fisher Scientific). Then, the cells were incubated with Annexin V conjugated to Alexa488 (A13201, Thermo Fisher Scientific) for 20 min, washed by the annexin-binding buffer, and were analyzed using the Attune NxT flow cytometer (Life Technologies). The data were analyzed using the Attune NxT Software (Life technologies).

### 2.10. TCGA Database Analysis

TCGA GTEX (healthy skin biopsies) and SKCM (primary and metastatic melanoma) studies were accessed via the USCS Xena platform [29]. For comparison of the ASICs and ENaCs expression in normal skin and melanomas, the data on the mRNA expression of the genes *ACCN2, ACCN1, ACCN3, ACCN4, SCNN1A,* and *SCNN1G* coding the ASIC1, ASIC2, ASIC3, ASIC4, α-ENaC, and γ-ENaC subunits, respectively, were downloaded from the GTEX and SKCM studies and analyzed by the GraphpadPrism software (GraphPad Software). Please note, that the genes coding the ASIC1 and ASIC2 subunits are designated as *ACCN2* and *ACCN1*, respectively. For analysis of the relationships between the ASICs/ENaCs mRNA expression and melanoma patients’ survival, patients with non-glabrous primary or metastatic melanoma were subdivided into two groups with the *ACCN2, ACCN1, ACCN3, ACCN4, SCNN1A*, and *SCNN1G* expression above or below median value. Survival curves were plotted according to the Kaplan–Meier method and compared using the log-rank test directly in the USCS Xena platform interface.

### 2.11. Statistical Analysis

Data are presented as mean ± SEM. Sample numbers (*n*) are indicated in the figure legends. No exclusion criteria were applied for experimental data. The data were analyzed using the one-way ANOVA with appropriate multiple comparisons post-hoc test, One-sample *t*-test, or two-tailed *t*-test as indicated in the figure legends. For linear regression curve comparison the GraphPad Prism 8.0 ANCOVA test was used. For comparison of patients’ survival with different ASICs and ENaCs expression log-rank test was used. Differences in the data were considered statistically significant at *p* < 0.05. Analysis was performed using the GraphPad Prism 8.0 software (GraphPad Software).

## 3. Results

### 3.1. Acidification of Cell Environment Enhances Proliferation, Migration, and Invasion of Metastatic Melanoma Cells

No data about acidification influence on progression of metastatic melanoma cells were available previously. Here, we analyzed the influence of the cell media acidification on viability, morphology, growth, migration, and invasion of patient-derived metastatic tumorigenic mel P cells. Non-tumorigenic human Het-1A keratinocytes [30] were used as a model of normal non-transformed cells.

**Figure 1:**
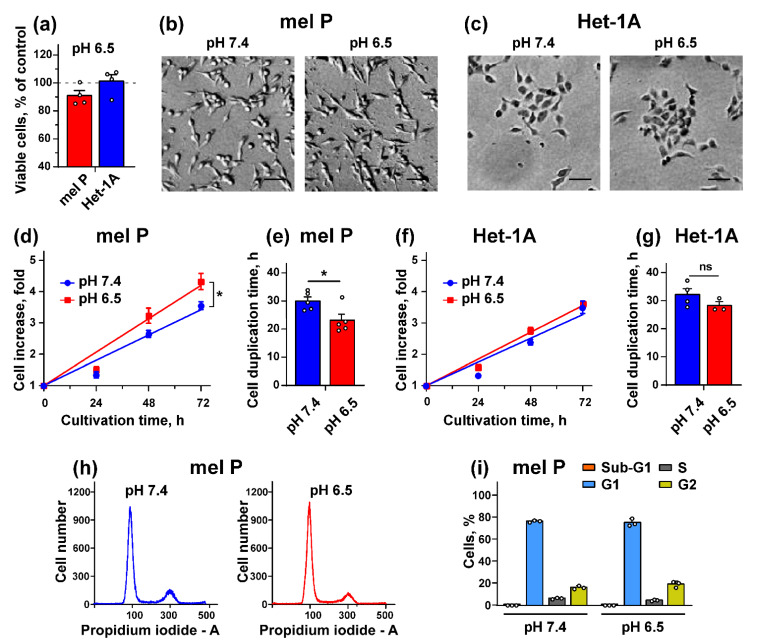
Effect of cell media acidification on growth of metastatic melanoma mel P cells and Het-1A keratinocytes. (**a**) Influence of cell media acidification on viability of mel P and Het-1A cells. Data were normalized to viability of cells cultivated at pH 7.4 (dashed line); (**b,c**) Influence of cell media acidification on morphology of mel P cells (**b**) and Het-1A cells (**c**), scale bar 25 µm; (**d**) Growth dynamics of mel P cells upon cultivation at pH 7.4 and 6.5 (*n* = 5). * (*p* < 0.05) indicates significant difference between regression line slopes by ANCOVA test; (**e**) Duplication time of mel P cells cultivated at pH 7.4 and 6.5 (*n* = 5). * (*p* < 0.05) indicates significant difference between data groups by two-tailed *t*-test; (**f**) Growth dynamics of Het-1A keratinocytes cultivated at pH 7.4 and 6.5 (*n* = 3–4); (**g**) Duplication time of Het-1A keratinocytes cultivated at pH 7.4 and 6.5 (*n* = 3–4); (**h**) Representative nuclei population distribution of mel P cells cultivated at pH 7.4 and 6.5; (**i**) % of cells in each cell cycle phase. Data are presented as % of cells in each cell cycle phase ± SEM (*n* = 3).

No significant influence of the cell media acidification from pH 7.4 to pH 6.5 during 96 h incubation on viability of mel P and Het-1A cells was revealed (Figure 1a). Morphology of the both types of cells also was not changed (Figure 1b,c). Next, we compared the growth parameters of mel P and Het-1A cells at normal (pH 7.4) and acidic (pH 6.5) conditions and found that proliferation of mel P cells cultivated at pH 6.5 during 96 h was significantly enhanced compared to the cells cultivated at pH 7.4 (Figure 1d). In line with it, the duplication time of mel P cells in acidic environment was lower by ~20% (Figure 1e). In contrast to mel P cells, the media acidification did not influence the growth parameters of Het-1A cells (Figure 1f,g). We did not find any influence of the media acidification on cell cycle progression in mel P cells (Figure 1h,i).

**Figure 2:**
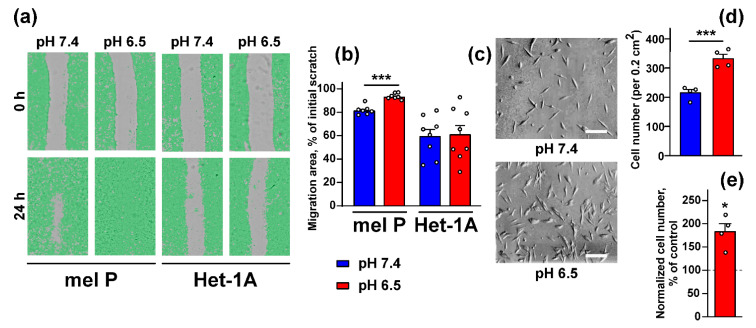
Influence of cell media acidification on mel P and Het-1A cell migration and invasion: (**a**) representative pictures of scratch test for mel P and Het-1A cells incubated at pH 7.4 and 6.5; (**b**) scratch square occupied by migrating mel P and Het-1A cells. Data are presented as % of the scratch surface, occupied by migrating cells ± SEM (*n* = 7–8), *** (*p* < 0.001) indicates significant difference between data groups by two-tailed *t*-test; (**c**) representative phase-contrast images showing mel P cells migrated through the 8 µM pore bottom of the migration chamber upon cultivation at pH 7.4 and 6.5 (×100 magnification, scale bar = 20 µm); (**d**) number of mel P cells migrated through the 8 µM pore bottom of the migration chamber upon cultivation at pH 7.4 and6.5. Data presented as the number of cells per 0.2 cm^2^ ± SEM (*n* = 4). *** (*p* < 0.001) indicates significant difference between data groups by two-tailed *t*-test; (**e**) number of migrated mel Pcells cultivated at pH 6.5 assayed by the WST-1 test and normalized to the number of migrated cells, cultivated at pH 7.4 (control, shown by dashed line). Data are normalized cell number ± SEM (*n* = 4). * (*p* < 0.05) indicates significant difference from the control by One-sample *t*-test.

The scratch assay, used here for investigation of cell migration, showed that the media acidification significantly increased migration of mel P cells, but not of Het-1A keratinocytes (Figure 2a,b). It should be noted that Het-1A cells were less mobile, in general, than mel P cells (Figure 2a,b). Cell invasion test with 8-µm pore migration chambers revealed that the number of migrated mel P cells was significantly higher when they were cultivated at pH 6.5 in comparison to the cells grown at pH 7.4 (Figure 2c,d), which was also confirmed by accounting of the migrated cells using the WST-1 assay (Figure 2e).

### 3.2. Acidification of Cell Environment Up-Regulates Expression of the ASIC1a, α-ENaC, and γ-ENaC Subunits in Metastatic Melanoma Cells

**Figure 3:**
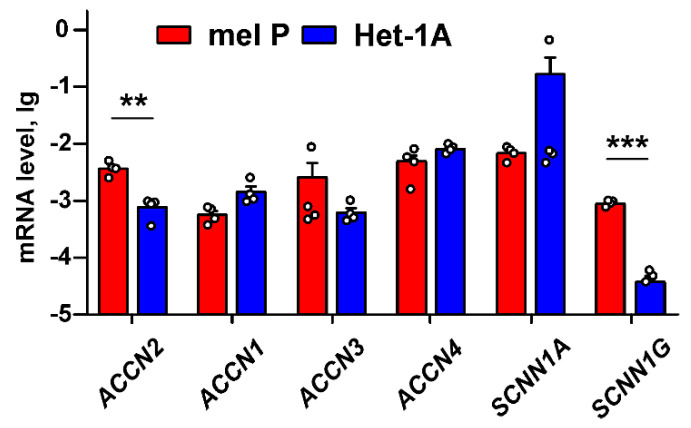
qPCR analysis of the *ACCN2*, *ACCN1*, *ACCN3*, *ACCN4*, *SCNN1A,* and *SCNN1G* expression in the patient-derived mel P melanoma cells and keratinocytes Het-1A. Gene expression was normalized to the β*-ACTIN, GPDH,* and *RPL13a* housekeeping genes and presented as lg of relative mRNA level ± SEM (*n* = 4). ** (*p* < 0.01) and *** (*p* < 0.001) indicate significant difference between data groups according to two-tailed *t*-test.

To explain the observed different influence of the acidification on growth and migration of mel P and Het1-A cells (Figure 1 and Figure 2), we proposed, that the cell media acidification can be related with an expression of different acid-sensitive channels from the DEG/ENaC family. Indeed, analysis of expression of the genes *ACCN2*, *ACCN1*, *ACCN3*, *ACCN4*, *SCNN1A,* and *SCNN1G* coding the ASIC1a, ASIC2, ASIC3, ASIC4, α-ENaC, and γ-ENaC subunits, respectively, in mel P cells and Het-1A keratinocytes revealed that the *ACCN2* and *SCNN1G* expression is significantly up-regulated in the cancer cells (Figure 3).

**Figure 4:**
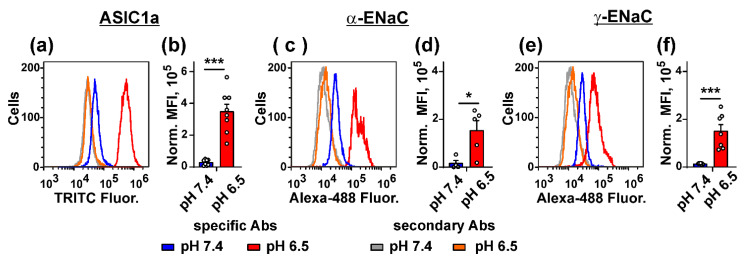
Influence of media acidification on expression of the ASIC1a (**a,b**), α-ENaC (**c,d**), and γ-ENaC (**e,f**) subunits in mel P cells: representative cell distribution histograms of the ASIC1a (**a**), α-ENaC (**c**), and γ-ENaC (**e**) subunits stained on the surface of mel P cells; expression levels of the ASIC1a (**b**), α-ENaC (**d**), and γ-ENaC (**f**) subunits. Data presented as normalized MFI ± SEM (*n* = 7–8). * (*p* < 0.05) and *** (*p* < 0.001) indicate significant difference between data groups by two-tailed *t*-test.

ASIC1a subunit (coded by the *ACCN2* gene) forms a heterotrimeric complex with the α-ENaC and γ-ENaC subunits in glioblastoma (GBM) cells [31]. We studied the influence of the cell media acidification on expression of the ASIC1a, α-ENaC, and γ-ENaC subunits in mel P cells by flow cytometry. Cell media acidification from pH 7.4 to pH 6.5 led to the drastic increase of the ASIC1a, α-ENaC, and γ-ENaC expression on a surface of mel P cells up to ~12-fold (Figure 4).

### 3.3. ASIC1a Inhibitor Mambalgin-2 Reduces Growth, Migration, and Invasion of Metastatic Melanoma Cells

As melanoma cells express ASIC1a (Figure 3), which is up-regulated upon cell media acidification (Figure 4), we tested the effect of the recombinant analog of mambalgin-2 from *Dendroaspis polylepis* on growth, migration, and invasion of mel P and Het-1A cells.

**Figure 5:**
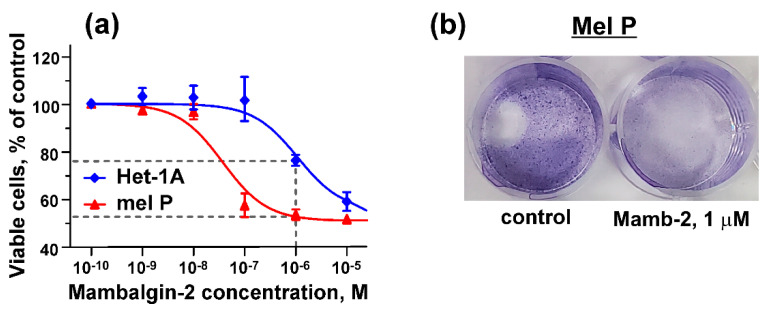
Influence of mambalgin-2 on growth of mel P and Het-1A cells: (**a**) effect of different mambalgin-2 concentrations on Het-1A and mel P cells cultivated at pH 6.5. The parameters describing the concentration-effect curves (EC_50_, A_1_) are: 37,3 ± 1.3 nM and 1009 ± 170 nM, 51.4 ± 2.3% and 55.4 ± 6.2%, for mel P and Het1-a cells, respectively. Data are presented as% of the control (untreated cells) ± SEM (*n* = 4). The difference between EC_50_ of the mambalgin-2 effect on mel P and Het-1A cells is statistically significant by F test (*p* < 0.001, *n* = 4); (**b**) picture illustrating the colony formation by mel P cells in absence (control) or presence of 1 µM mambalgin-2 according to the crystal violet assay.

WST-1 assay revealed that mambalgin-2 reduced proliferation of both mel P and Het-1A cells cultivated in the acidic media in the concentration-dependent manner with the similar maximal inhibitory effect (51.4 ± 2.3% and 55.4 ± 6.2%, respectively, Figure 5a), while EC_50_ of mambalgin-2 at cancer and non-malignant cells was significantly different (37.3 ± 1.3 nM and ~1009 ± 170 nM for mel P and Het-1A cells, respectively, Figure 5a).

As a result, the maximal inhibition effect of mambalgin-2 on mel P cells was observed at 1 µM concentration, while the maximal inhibition effect of the toxin on Het1-A keratinocytes was observed at concentrations much more than 10 µM. For comparison, the effects of 1 µM mambalgin-2 on mel P and Het1-A cells are shown by dashed lines (Figure 5a). In line with the data obtained, crystal violet assay showed that mambalgin-2 dramatically inhibited the colony formation by mel P cells (Figure 5b).

**Figure 6:**
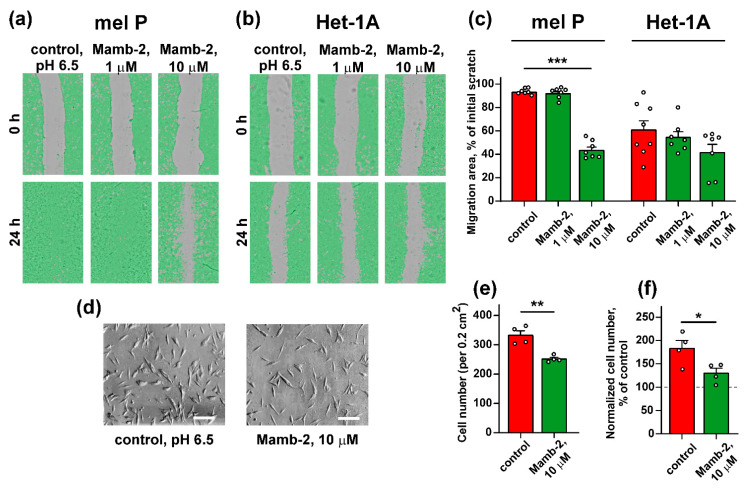
Influence of mambalgin-2 on mel P and Het-1A cell migration and invasion: (**a,b**) representative pictures of scratch test for mel P (**a**) and Het-1A (**b**) cells incubated at pH 6.5 in absence or presence of mambalgin-2; (**c**) scratch square occupied by migrating mel P and Het-1A cells incubated at pH 6.5 in absence or presence of mambalgin-2. Data are presented as % of the scratch surface, occupied by migrating cells ± SEM (*n* = 7–8), *** (*p* < 0.001) indicates significant difference between data groups by One-Way ANOVA followed by Tukey’s post hoc test; (**d**) representative phase-contrast images showing mel P cells migrated through the 8 µM pore bottom of the migration chamber upon cultivation at pH 6.5 in absence or presence of mambalgin-2 (× 100 magnification, scale bar = 20 µm); (**e**) number of mel P cells migrated through the 8 µM pore bottom of the migration chamber upon cultivation at pH 6.5 in absence or presence of mambalgin-2. Data presented as the number of cells per 0.2 cm^2^ ± SEM (*n* = 4). ** (*p* < 0.01) indicates significant difference from the data groups by two-tailed *t*-test; (**f**) number of migrated mel P cells cultivated at pH 6.5 in absence or presence of mambalgin-2 assayed by the WST-1 test and normalized to the number of migrated cells cultivated at pH 7.4 in absence of mambalgin-2 (shown by dashed line). Data are normalized cell number ± SEM (*n* = 4). * (*p* < 0.05) indicates significant difference between the data groups by two-tailed *t*-test.

Similar to proliferation, the mambalgin-2 effect on mel P cells motility was dose-dependent. Application of 1 µM mambalgin-2 did not change migration of cancer cells, while mambalgin-2 at 10 µM concentration down-regulated the migration area more than 2-fold as compared to the control (untreated cells, Figure 6a,c). At the same time, no influence of mambalgin-2 on motility of Het-1A keratinocytes was found up to the toxin’s concentration of 10 µM (Figure 6b,c). The cell invasion test revealed that the application of 10 µM mambalgin-2 to mel P cells cultivated in the acidic media significantly reduced the number of migrated cells (Figure 6d,e), that was also confirmed by the WST-1 assay (Figure 6f).

### 3.4. Mambalgin-2 Induces Apoptosis in Mel P Cells

**Figure 7:**
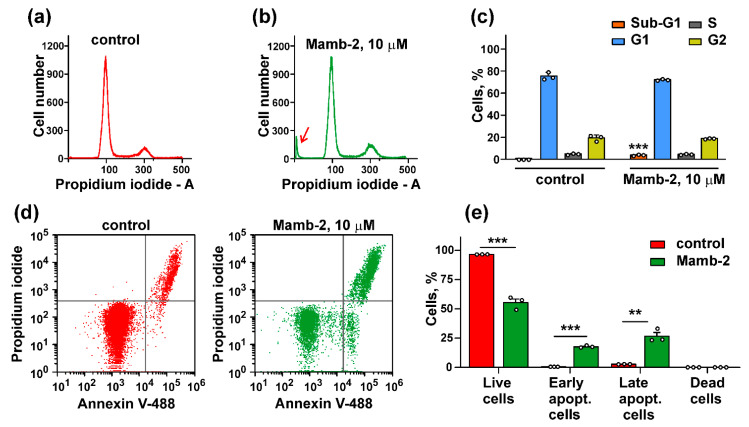
Apoptosis induction by mambalgin-2 in mel P cells: (**a,b**) Representative nuclei population distribution of mel P cells cultivated at pH 6.5 in absence (control, **a**) or presence of 10 µM mambalgin-2 (**b**). Peak corresponding to the Sub-G1 cell population is shown by red arrow; (**c**) % of cells in each cell cycle phase. Data are presented as % of cells in each cell cycle phase ± SEM (*n* = 3). *** (*p* < 0.001) indicates significant difference between the sub-G1 nuclei groups by two-tailed *t*-test; (**d**) representative pictures of phosphatidylserine externalization analysis upon the 10 µM mambalgin-2 treatment of mel P cells by flow cytometry with Annexin V-488 and Propidium iodide (control is without mambalgin-2); (**e**) percentage of mel P cells with externalized phosphatidylserine and bound propidium iodide in absence (control) or presence of 10 µM mambalgin-2. The data are presented as % of live, early apoptotic, late apoptotic and dead cells ± SEM (*n* = 3). ** (*p* < 0.01) and *** (*p* < 0.001) indicate the significant difference between data groups by a two-tailed *t*-test.

We did not observe any effect of 10 μM mambalgin-2 on the cell cycle progression in mel P cells cultivated at pH 6.5 (Figure 7a–c), but found the formation of the sub-G1 peak, which is characteristic for apoptosis [32]. To further justify the apoptosis induction in mel P cells upon the mambalgin-2 treatment, we used the Annexin V/Propidium iodide assay. The analysis by flow cytometry revealed that the number of mel P cells with externalized phosphatidylserine significantly increased upon the incubation with 10 μM mambalgin-2 from ~0.5% to ~18% (Figure 7d,e). Moreover, ~27% of mel P cells possessed not only externalized phosphatidylserine, but also bound propidium iodide upon the mambalgin-2 treatment (Figure 7d,e). This points to the membrane integrity loss and late apoptosis induction, which is consistent with an appearance of the sub-G1 cell population on the cell cycle histogram (Figure 7b,c).

### 3.5. Mambalgin-2 Inhibits Expression and Activation of Melanoma Progression Markers in Mel P Cells

**Figure 8:**
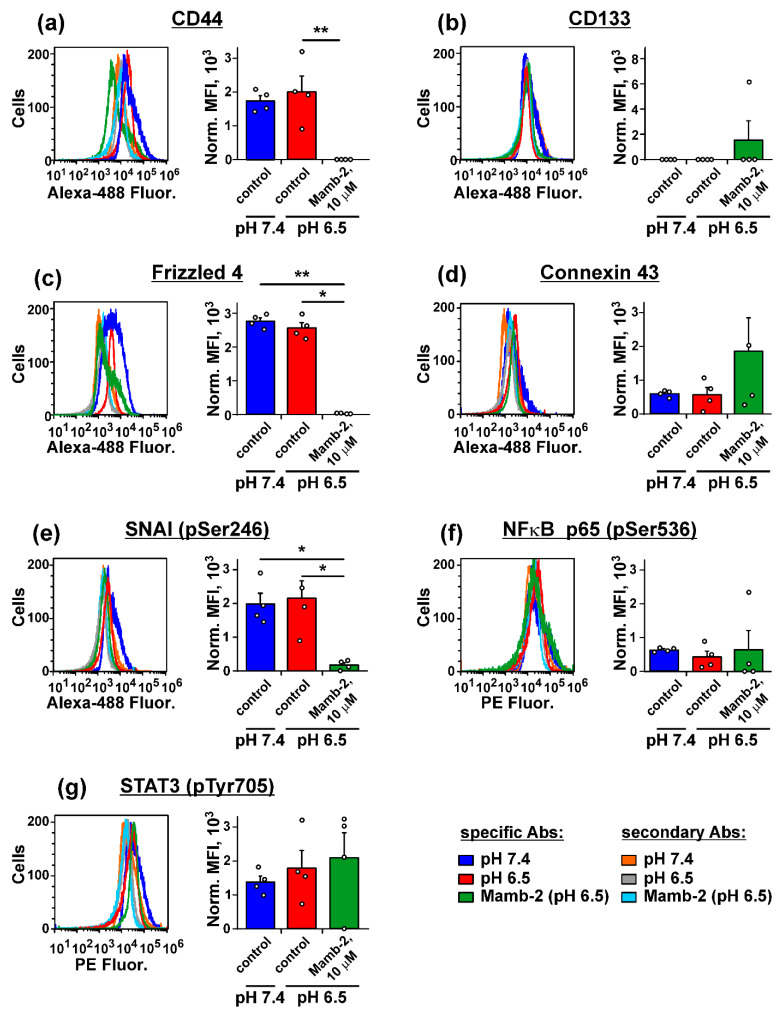
Analysis of influence of cell media acidification (96 h) and 10 µM mambalgin-2 (72 h) on expression and activity of molecules mediating progression in mel P cells: CD44 (**a**), CD133 (**b**), Frizzled 4 (**c**), connexin 43 (**d**), SNAI (pSer246) (**e**), NFκB p65 (pSer536) (**f**), and STAT3 (pTyr705) (**g**). Analysis was performed by flow cytometry. The representative cell distribution histograms after staining by antibodies and expression levels are shown at the left and right figure panels, respectively. Data presented as normalized MFI ± SEM (*n* = 4). * (*p* < 0.05) and ** (*p* < 0.01) indicate significant difference between the data groups by One-Way ANOVA followed by Tukey’s hoc test. Cells stained only by secondary antibodies (Abs) were used as a negative control.

Besides ASICs and ENaCs, adaptation of melanoma and other cancer cells to fast growth and related to it acidic environment can be connected with an activity of other receptors and intracellular messengers, that provide stemness (CD44 [33], CD133 [34]), growth, migration, and senescence regulation (Frizzled 4 [35,36]), extirpation of extracellular lactate (connexin 43 [37]), stroma remodeling, invasion, apoptosis resistance as well as gene transcription regulation (SNAI [38], NFκB [39], and STAT3 [40]). The flow cytometry-based assay revealed that expression of CD44 and Frizzled 4, as well as phosphorylation of SNAI at Ser246 were significantly reduced in mel P cells upon incubation with 10 μM mambalgin-2 (Figure 8a,c,e). However, mambalgin-2 did not influence expression of CD133 and connexin 43, and phosphorylation of NFκB p65 (pSer536) and STAT3 (pTyr705) in mel P cells (Figure 8b,d,f,g). At the same time, no changes in expression or phosphorylation of the investigated molecules were found upon the cell incubation at neutral and acidic pH in absence of mambalgin-2 (Figure 8).

### 3.6. Mambalgin-2 Interacts with ASIC1, α-ENaC, and γ-ENaC on the Cell-Surface Membrane of Mel P Cells

Mambalgin-2 is known to inhibit the ASIC1a activity in *Xenopus laevis* oocytes and in tumor cells [10,23]. From the other hand, the ASIC1a, α-ENaC, and γ-ENaC subunits can form heterotrimers in GBM cells [31], and expression of these subunits was up-regulated upon acidification in mel P cells (Figure 4). We hypothesized that, similar to GMB cells, melanoma cells also can express the heterotrimers formed by these subunits, and mambalgin-2 can target it.

**Figure 9:**
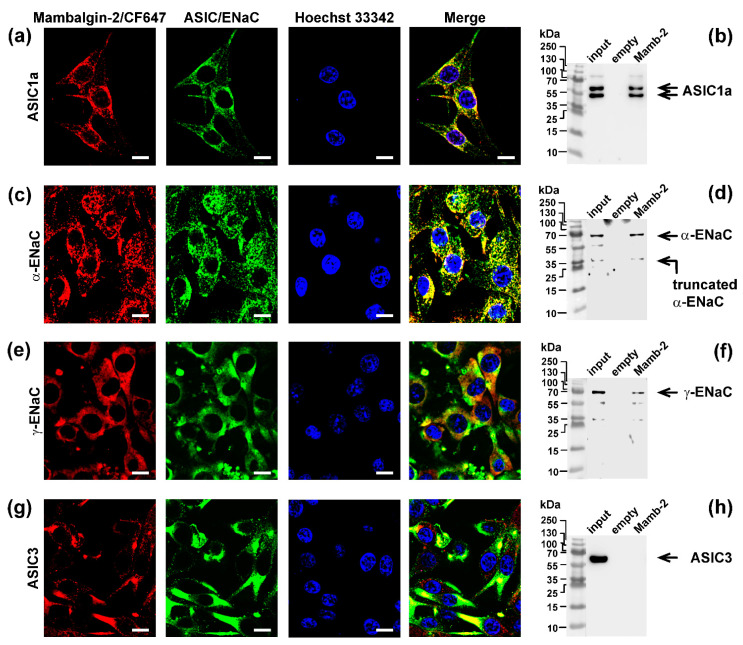
Investigation of mambalgin-2 interaction with the ASIC1a (**a**,**b**), α-ENaC (**c**,**d**), γ-ENaC (**e**,**f**), and ASIC3 (**g**,**h**) subunits in mel P cells: localization of fluorescently-labelled mambalgin-2/CF647 (red) and Alexa488-labeled ASIC1 (**a**), α-ENaC (**c**), γ-ENaC (**e**), and ASIC3 (**g**) subunits (green). Nuclei were stained by Hoechst 33342 (blue), scale bar 10 µm. Western blot analysis of the ASIC1 (**b**), α-ENaC (**d**), γ-ENaC (**f**), and ASIC3 (**h**) subunits extraction from membrane fraction of mel P cells by affinity chromatography on NHS-sepharose resin coupled with mambalgin-2 (*n* = 3).

Indeed, study of localization of fluorescently-labeled mambalgin-2/CF647 revealed the selective colocalization of mambalgin-2 with the ASIC1a, α-ENaC, and γ-ENaC subunits, but not with ASIC3 in mel P cells (Figure 9a,c,e,g). Notably, the major amount of ASIC3 was predominantly located inside cells (Figure 9g).

ASIC1a, α-ENaC, and γ-ENaC were colocalized with mambalgin-2/CF647 almost in all areas. However, the staining patterns of mambalgin-2/CF647 and ASIC3 were not correlated. Some cells and membrane regions with high ASIC3 expression did not bind mambalgin-2/CF647, while the regions with high affinity for mambalgin-2/CF647 did not contain ASIC3. Although these results cannot rule out absence of interaction between mambalgin-2/CF647 and ASIC3, they clearly indicate that ASIC3 alone was not sufficient to bind mambalgin-2.

To confirm the direct interaction of mambalgin-2 with ASIC1a, α-ENaC, and γ-ENaC, we performed an extraction of the ASIC1a, ASIC3, α-ENaC, and γ-ENaC subunits from a membrane fraction of mel P cells by affinity chromatography using an N-hydroxysuccinimide resin coupled with mambalgin-2. The empty resin blocked by 500 mM ethanolamine was used as a negative control. Consistent with the confocal microscopy, mambalgin-2 extracted detectable amounts of the ASIC1a, α-ENaC, and γ-ENaC subunits, but not of ASIC3 (Figure 9b,d,f,h). Altogether, our data point to the direct interaction of mambalgin-2 with ASIC1a, α-ENaC and γ-ENaC on the membrane of the mel P cells.

**Figure 10:**
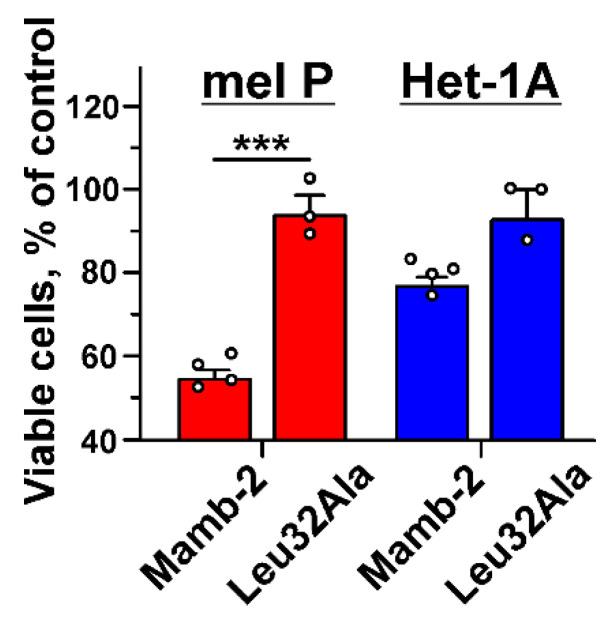
Influence of mambalgin-2 and its Leu32Ala mutant on proliferation of mel P and Het-1A cells. Toxins’ concentration in the cell media was 1 μM. Data are presented as % of the control (untreated cells, dashed line) ± SEM (*n* = 3–4). *** (*p* < 0.001) indicates significant difference in the activity of mutant and mambalgin-2 according to two-tailed *t*-test.

Previously it was revealed that the central loop of mambalgin molecule is the main binding site for interaction with the ASIC1a channel [41]. To study the role of this loop in the interaction of mambalgin-2 with the heterocomplex ASIC1a/α-ENaC/γ-ENaC in mel P cells, we used the variant of mambalgin-2 with the mutation Leu32Ala, which leads to reduced inhibitory activity of the toxin towards ASIC1a [42]. Contrary to the wild-type toxin, mutant did not inhibit proliferation of mel P cells and demonstrated diminished antiproliferative activity on Het1-A cells (Figure 10), pointing on the same binding site of mambalgin-2 with the ASIC1a and ASIC1a/α-ENaC/γ-ENaC channels. Thus, the ASIC1a subunit is the primary target of mambalgin-2 in mel P and Het1-A cells.

### 3.7. Up-Regulation of the ACCN2 Expression in Patients with Metastatic Melanoma Correlates with Poor Survival Prognosis

**Figure 11:**
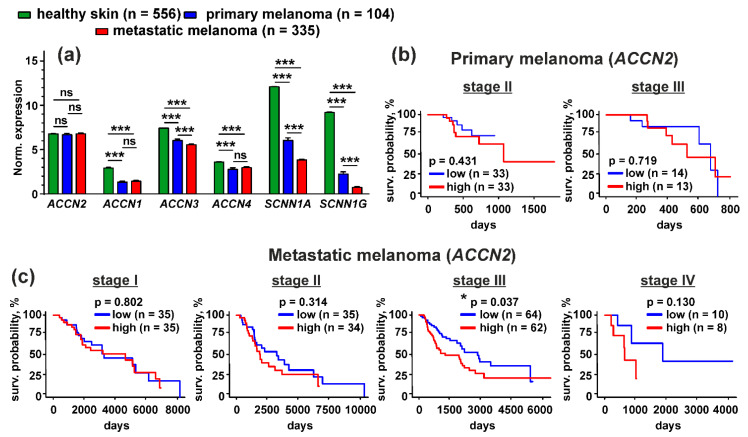
Bioinformatic analysis of the *ACCN2*, *ACCN1*, *ACCN3*, *ACCN4*, *SCNN1A,* and *SCNN1G* expression in healthy people and patients with non-glabrous primary and metastatic melanoma from the GTEX and TCGA SKSM databases: (**a**) analysis of the gene expression in healthy skin biopsies (GTEX study) and in samples of primary and metastatic melanoma (TCGA SKSM study). Data are presented as normalized gene expression ± SEM, *** (*p* < 0.001) indicates significant difference between the data groups by One-Way ANOVA followed by Dunnets’ hoc test; (**b**,**c**) Kaplan–Meier analysis of survival of the patients with primary (**b**) and metastatic (**c**) melanoma and different expression of the *ACCN2* gene. * (*p* < 0.05) indicates significant difference between the survival prognosis for patients with the high (above median) and low (below median) *ACCN2* expression according to log-rank test.

To evaluate the physiological role of up-regulation of expression of different members of the DEG/ENaC family in melanoma cells, we performed a comparative analysis of expression of the *ACCN2*, *ACCN1*, *ACCN3*, *ACCN4*, *SCNN1A,* and *SCNN1G* genes in non-glabrous primary and metastatic melanoma cells and in human healthy skin samples from the TCGA SKSM and GTEX databases. We found that the *ACCN2* expression in healthy skin and in primary and metastatic melanoma lesions did not differ significantly, while the *ACCN1*, *ACCN3*, *ACCN4*, *SCNN1A,* and *SCNN1G* expression was dramatically down-regulated in primary and metastatic melanoma samples compared to normal skin biopsies (Figure 11a). Even more decrease of the *ACCN3*, *SCNN1A,* and *SCNN1G* expression was observed in comparison of primary malignancies and metastatic melanoma samples. As a result, the gene coding the ASIC1a subunit became overexpressed in comparison with the genes coding other ASIC and ENaC subunits both in primary and metastatic melanomas (Figure 11a).

To study a correlation between the *ACCN2* expression level and survival prognosis for patients with primary and metastatic melanomas, we performed Kaplan-Meier analysis of the patient biopsies from the TCGA SKCM database. *ACCN2* expression did not correlate with the survival prognosis for the patients with primary melanoma at stages II and III (Figure 11b). Analysis of the samples from the patients with primary melanoma at stages I and IV was not performed due to a low number of the patients (*n* = 2 and 3, respectively). However, the analysis of the metastatic melanoma dataset showed that the patients with the disease at stage III and with lower expression of the *ACCN2* gene demonstrated better survival prognosis than the patients with the same stage of metastatic melanoma and with the elevated *ACCN2* expression (Figure 11c). The same relationship was observed for the patients with metastatic melanoma at stage IV, but it did not reach the statistical significance (Figure 11c). Expression of all other investigated genes in metastatic melanoma did not correlate with the patient survival prognosis (data not shown).

## 4. Discussion

The extracellular media acidification is characteristic for many cancers including melanoma [5,43], and adaptation of cancer cells to acidosis includes changes in protein transcription and post-translational modifications, as well as changes in cancer cell morphology, growth, and motility [44]. Media acidification inhibits viability and cell cycle progression and induces stem-like cell phenotype in primary A375 melanoma cells [8], while it drives growth, migration, and invasion of breast, lung and hepatocellular cancers, as well as of highly invasive C8161 and poorly invasive A375P melanoma cells [5,6,7,13,45,46]. Here we investigated the influence of the acidic environment on morphology, growth, migration, and invasion of metastatic melanoma cells and normal keratinocytes. In contrast to primary melanoma cells [7,8,45], the media acidification did not influence the viability, morphology, and cell cycle progression of the metastatic melanoma cells and normal keratinocytes, although mel P cells preincubated in the acidic media started to grow more rapidly with a consequent decrease of cell duplication time (Figure 1). Together with the stimulating effect of the cell media acidification on migration and invasion (Figure 2), these point on good adaptation of mel P cells to the acidic environment.

To date, possible involvement of proton sensors in the adaptation of melanoma cells to the acidic environment and their implication in melanoma progression remained poorly explored. Only ASIC1 and ASIC2 were shown to be expressed in human melanoma and skin cancer tissues previously [16]. Here, for the first time, we performed extensive screening of expression of the members of the DEG/ENaC family, responsible for regulation of extracellular acidification, in normal keratinocytes and metastatic melanoma cells. Overexpression of the ASIC1a, α-ENaC, and γ-ENaC subunits in cancer cells, as well as upon the cell media acidification was revealed (Figure 3 and Figure 4). In line with the data obtained, the media acidification was shown to up-regulate the ASIC1 expression in breast [47] and colorectal [48] cancer cells.

Media acidification increases metastatic melanoma cell proliferation, migration, and invasion (Figure 1 and Figure 2) and up-regulates the ASIC1a subunit expression (Figure 3 and Figure 4). In turn, ASIC1 knock-down inhibits migration of breast cancer cells induced by the cell media acidification [49]. Thus, targeting of ASIC1a may be a promising strategy to control growth and migration of melanoma cells. To test this possibility, we used the recombinant analog of the effective and selective ASIC1a inhibitor—mambalgin-2. Recently, we have shown that mambalgin-2 inhibits the ASIC1a activity in GBM [23] and leukemia [10] cells, as well as cell proliferation. Indeed, incubation with mambalgin-2 significantly reduced viability of mel P and Het-1A cells preincubated at acidic pH (Figure 5). Notably, EC_50_ of mambalgin-2 was significantly lower in melanoma cells in comparison to Het-1A keratinocytes (~ 37 and 1000 nM, respectively), pointing on presence of the “pharmacological window”—the concentration range in which mambalgin-2 can inhibit growth of cancer cells without toxicity to normal ones. The implication of ASIC1a in EMT was shown for GBM, lung, breast and other cancer cells [5,12,14,15,19], however, the influence of ASIC1a targeting on migration and invasion of melanoma cells remained unclear. In line with the data on cell proliferation, mambalgin-2 inhibited migration and invasion of mel P cells induced by the cell media acidification (Figure 6). Moreover, we showed that mambalgin-2 induced apoptosis of metastatic melanoma cells (Figure 7). We showed previously that apoptosis induction underlies the mambalgin-2 antiproliferative action in GBM cells [23], and propose here that the growth inhibition due to the apoptosis induction is common for the mambalgin-2 mediated ASIC1 inhibition in cancer cells.

Investigation of the molecular mechanisms underlying the mambalgin-2 activity in metastatic melanoma cells and their adaptation to the cell media acidification revealed that incubation of mel P cells with mambalgin-2 leads to the down-regulation of CD44 and Frizzled 4 cell-surface expression (Figure 8a,c). CD44, usually activated by hyaluronan, controls cell proliferation, survival, cytoskeleton assembly and enhances cellular motility [49]. High CD44 surface expression in primary melanoma correlates with increased metastatic risk and reduced survival [33]. Interestingly, CD44 interacts with the Na^+^/H^+^ exchanger NHE1 and participates in acidification-induced EMT in breast cancer cells [50]. We propose that the down-regulation of CD44 expression may also inhibit its possible interaction with NHE1 in melanoma cells that may lead to dysregulation of intracellular pH balance. Frizzled 4 activates the WNT/β-catenin signaling in many cancer cells [51] and its down-regulation leads to inhibition of bladder cancer cell migration [52]. In liver cancer cells, the ASIC1a activation promotes proliferation via the β-catenin activation [53], thus, inhibition of the ASIC1a activity by mambalgin-2 could also inhibit the WNT/β-catenin signaling in melanoma cells. Mambalgin-2 treatment also inhibited phosphorylation of the transcription factor SNAI (pSer246) (Figure 8e), which drives EMT in melanoma and carcinoma cells [54,55,56]. Moreover, ASIC1 promotes acidosis-induced expression of SNAI, that in turn leads to invasion of prostate cancer cells [57]. Absence of the changes in CD133 and connexin 43 expression, as well as in phosphorylation of the transcription factors NFκB p65 (pSer536) and STAT3 (pTyr705) in mel P cells treated by mambalgin-2 (Figure 8b,d,f,g) indicates that the toxin effect is not linked with the lactate extirpation from the cells and is not mediated by gene transcription regulation by NFκB p65 and STAT3. Notably, no changes in the expression/phosphorylation of the investigated messengers were found upon acidification of the cell media from pH 7.4 to pH 6.5 in absence of mambalgin-2 (Figure 8). Thus, adaptation of mel P cells to the extracellular media acidification is not provided by CD44, Frizzled 4 or SNAI, but correlates with the function of acid-sensing channels.

GBM cells, unlike healthy astrocytes or low-stage glioma cells, express heterotrimeric hybrid channels composed of the ASIC1a, α-ENaC, and γ-ENaC subunits. Formation of these hybrid channels is observed upon the cell media acidification [31] and drives GBM growth and progression [11,31,58]. Simultaneous up-regulation of expression of the ASIC1a, α-ENaC, and γ-ENaC subunits on the cell-surface membrane of mel P cells upon the cell media acidification (Figure 4) allowed us to hypothesize that the target of mambalgin-2 in metastatic melanoma cells are the heterotrimeric hybrid channels ASIC1a/α-ENaC/γ-ENaC rather than homomeric channels ASIC1a. Indeed, confocal microscopy together with the data of affinity extraction confirmed this proposal (Figure 9). The fact that the mambalgin-2 mutant with impaired activity towards the ASIC1a channels [42] demonstrates reduced antiproliferative activity on mel P and Het1-A cells (Figure 10), possibly points on the similar binding site of mambalgin-2 at the homomeric ASICa and heteromeric ASIC1a/α-ENaC/γ-ENaC channels. Analysis of the cryo-EM structure of the complex of rat ASIC1a with mambalgin-1 (having one amino acid substitution beyond the active site in comparison with mambalgin-2) revealed that the toxin interacts mainly with the thumb of the ASIC1a subunit and does not bind the receptor at the interface of subunits [41]. Similar to that, mambalgin-2 also can interact mainly with the ASIC1a subunit as a part of the heterocomplex ASIC1a/α-ENaC/γ-ENaC. Notably, mambalgin-2 demonstrates much higher EC_50_ of mambalgin-2 at keratinocytes in comparison with mel P cells. The difference in EC_50_ can be connected with the lower ASIC1a and γ-ENaC mRNA expression levels in Het-1A cells in comparison with mel P cells (Figure 3), which in turn can result in the lower presentation of the homomeric and/or heteromeric channels on the cell surface of normal keratinocytes. Similar reduced expression of ASIC1a mRNA was reported for normal astrocytes in comparison with glioma cells [23]. Thus, the expression level of ASIC1a may serve as a marker of cell sensitivity to mambalgin-2.

To evaluate the physiological relevance of the ASICs and ENaC channels in melanoma, we performed the analysis of the TCGA database and revealed that expression of ASIC2, ASIC3, ASIC4, α- and γ-ENaC but not of ASIC1 mRNA was significantly down-regulated in samples of patients with primary and metastatic melanoma compared to normal skin biopsies (Figure 11a). As a result, ASIC1 expression in melanoma lesions is relatively elevated in comparison to other acid-sensitive channel subunits that may point on the possible ASIC1 participation in melanoma progression. The bioinformatic analysis confirmed this observation, as patients with metastatic melanoma and with lower expression of the *ACCN2* gene demonstrate a better survival prognosis than patients with the elevated *ACCN2* expression (Figure 11c). Thus, the ASIC1 expression level may serve as a prooncogenic marker too.

Generally, melanoma, as well as non-melanoma skin cancers are usually treated with surgery followed by immunotherapy or radiotherapy. High-dose rate radiotherapy was shown recently to be effective for treatment of non-melanoma skin cancer [59]. Peri-induction radiotherapy induces tumor cell death in the central hypoxic segment of tumor [60], stimulates anti-tumor immunity and regulates the tumor microenvironment by T cell activation [61,62]. ASICs targeting may provide an additional opportunity to control the melanoma microenvironment. Combined use of mambalgin-2 or its mimetics with radiotherapy or immunotherapy may become a new strategy for the complex therapy of melanoma by reducing the radiation dose and controlling growth and migration of melanoma cells.

## 5. Conclusions

For the first time, we showed that acidification of extracellular media drives proliferation, migration, and invasion of patient-derived metastatic melanoma cells, but not of normal keratinocytes. These processes are accompanied by the increase of the ASIC1a, α-ENaC, and γ-ENaC subunit expressions on the surface of metastatic melanoma cells. Targeting of the ASIC1a/α-ENaC/γ-ENaC heterocomplex by the selective inhibitor of the ASIC1a channels—mambalgin-2—cancels the prooncogenic processes, down-regulates the expression and activation of the messengers related with them, and induces apoptosis in metastatic melanoma cells. In addition, ASIC1 over-expression was found to be a marker of poor survival prognosis for the patients with metastatic melanoma. Thus, targeting of the ASIC1a-containing channels expressed on the cell surface by drugs such as mambalgin-2 can be considered as a promising strategy for the treatment of metastatic melanoma.

## Figures and Tables

**Table 1:** Primers, used for qPCR experiments.

Gene	Primer		Amplicon Size, bp
	Forward	Reverse	
*β-actin*	CATGTACGTTGCTATCCAGGC	CTCCTTAATGTCACGCACGAT	88
*GPDH*	ACAACTTTGGTATCGTGGAAGG	GCCATCACGCCACAGTTTC	73
*RPL13a*	TCAAAGCCTTCGCTAGTCTCC	GGCTCTTTTTGCCCGTATGC	104
*ASIC1a*	CGAAGCAGGCATCAAAGTGC	TTTGGATGATAGGGAGCCACG	642
*ASIC2*	CACCAAGACTTCACCACAGTGTTT	TGTAGCGGGTCTCACAGTCA	409
*ASIC3*	TACAGAACTGTGCCCACCC	GGTCTTCGGAACAGAGCAGA	502
*ASIC4*	GAGGAGAGAGACAAGCGGCA	GTCCAGCATGATCTCCAGGC	930
*α* *-ENaC*	CCAGGCCGCTGCACCT	GCCGATCTTCCAGTCCTTCC	750
*γ* *-ENaC*	GAGTGACGTGCCAATCAGGA	TCTCCGAAACCACAGATGGC	305

## Data Availability

Data generated within experiments is available on request.
